# Supplementary material for: Resource sharing in technologically defined social networks
Source: Nat Commun. 2019 Mar 6;10:1079. doi: 10.1038/s41467-019-08935-2 (PMC6403336; doi:10.1038/s41467-019-08935-2)
Supplement: Supplementary file 1 — Supplementary Information [file 41467_2019_8935_MOESM1_ESM.pdf]

Resource Sharing in Technologically Defined Social Networks

Shirado et al.

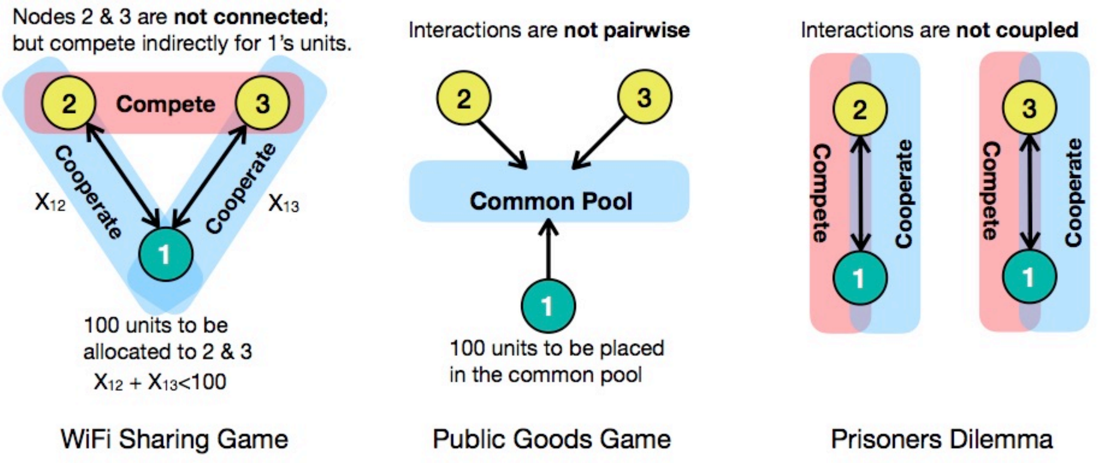

Supplementary Figure 1

Properties and Structure of WiFi Sharing Game. The WiFi sharing game has both a cooperative and a competitive structure unlike pure cooperative and pure competitive games. Furthermore, the decisions of the actors are made pairwise (unlike public good games) but are coupled with each other (unlike Prisoners Dilemma games).

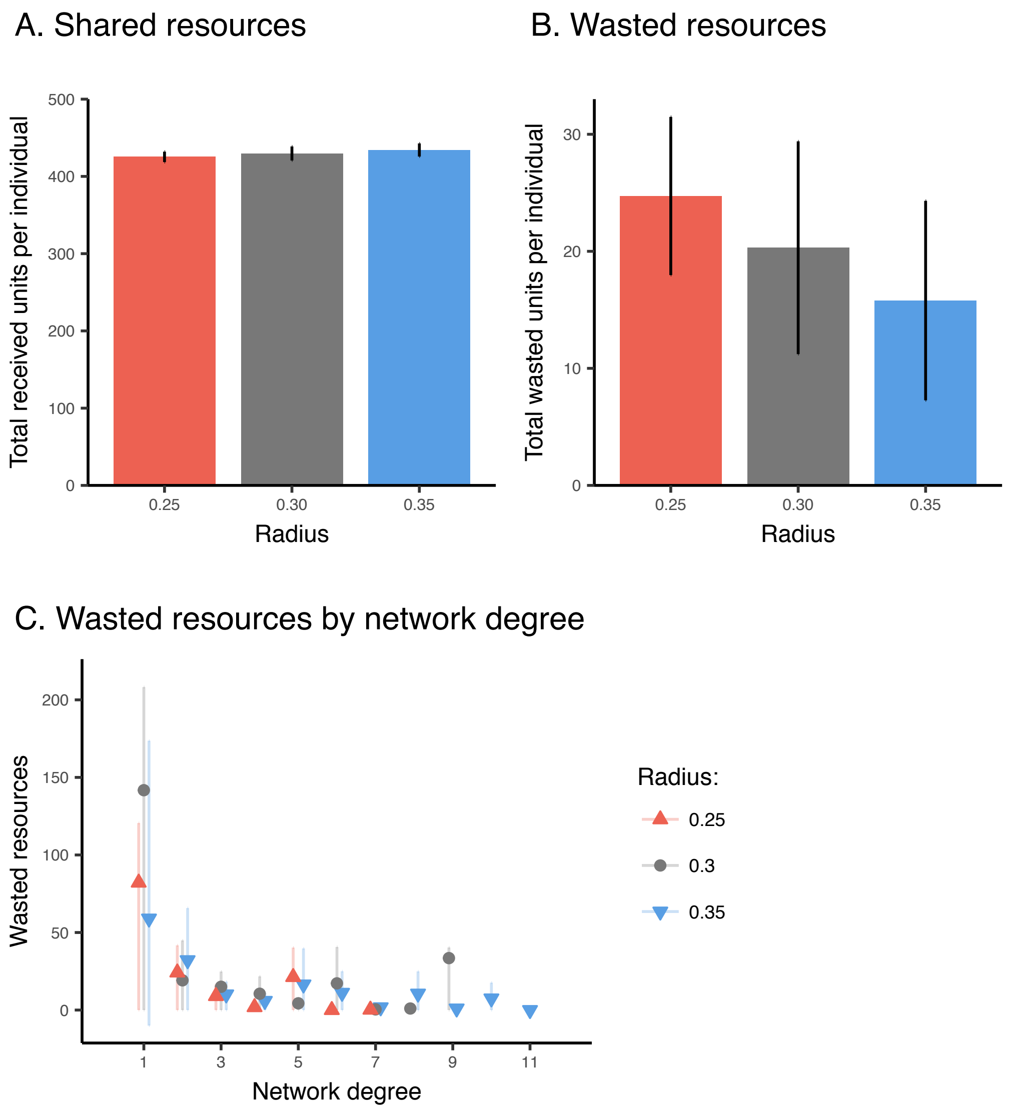

Supplementary Figure 2

(A) Final average wealth (i.e., total received units) and (B) wasted resources (i.e., total unallocated units) across the connection radii. Error bars are 95% confidence intervals (N=10 sessions per radius). The total wealth that all subjects in a session could access was a maximum of 15 rounds x 30 units = 450 units. While the amount of wasted resources decreases with increases in the connection radius (and so network density), individual wealth has no significant difference among the connection radii because very little of the resources are wasted compared to the total amount. (C) At the individual end, subjects having only one neighbor were especially likely to waste their resources.

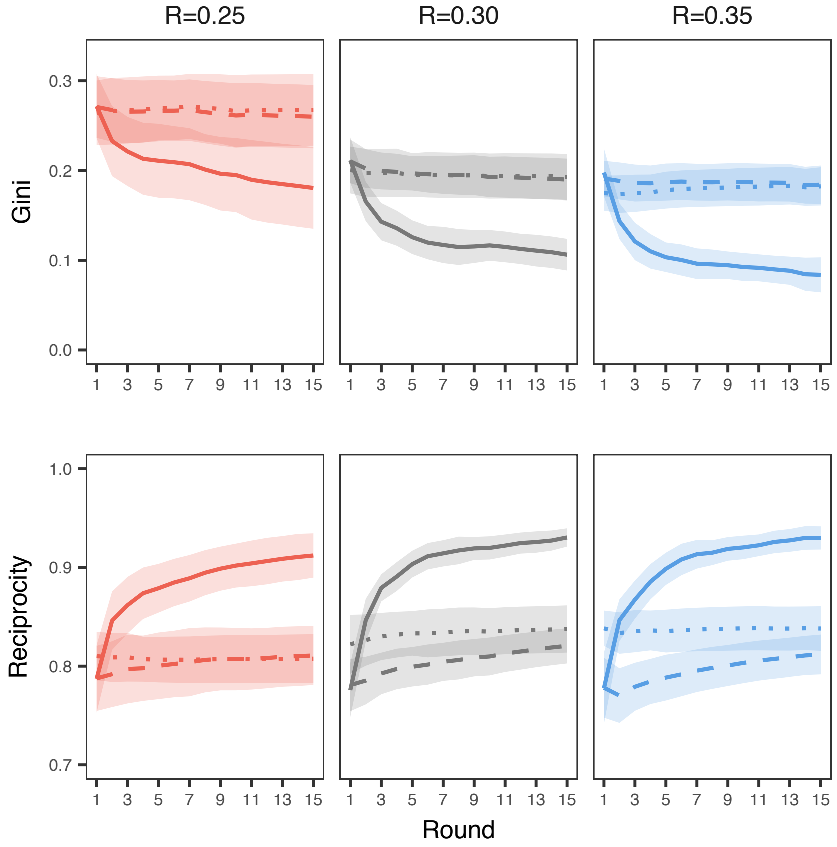

Supplementary Figure 3

Changes in Gini coefficient and network-level reciprocity across rounds. The experimental results (solid lines) show that human subjects reduce wealth inequality and increase reciprocity level with their neighbors over time, compared to null models representing only structural effects (dashed and dotted lines). We used two null models for comparison; in one, each individual randomly allocates their resources while keeping the diversity of the actual allocations from the experiment (dashed lines); and in the other, each individual equally allocates their resources to their neighbors (dotted lines). Shaded areas denote 95% confidence intervals (N=10).

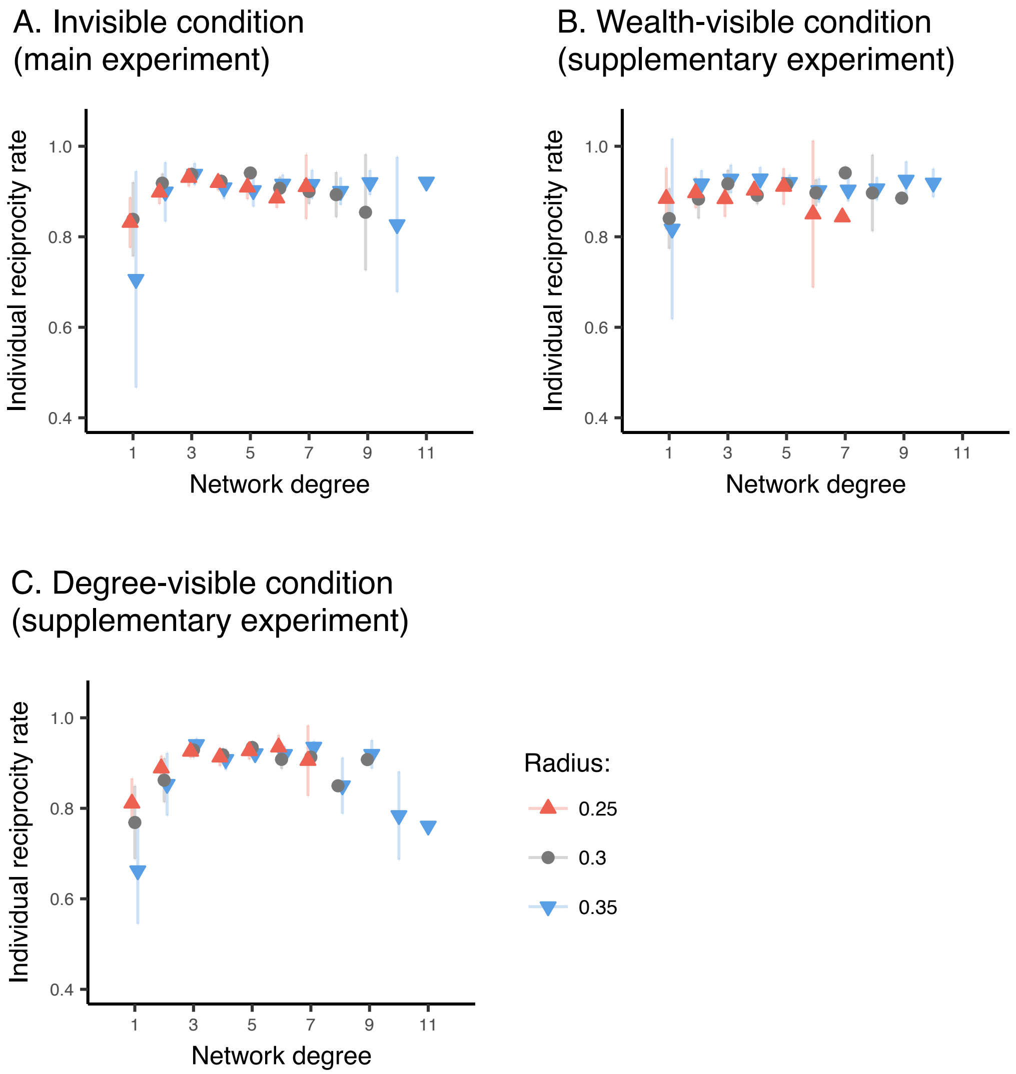

Supplementary Figure 4

Changes in individual-level reciprocity by network degree. Error bars denote 95% confidence intervals. "Individual reciprocity rate" is the average of symmetric transaction rate for each neighbor.

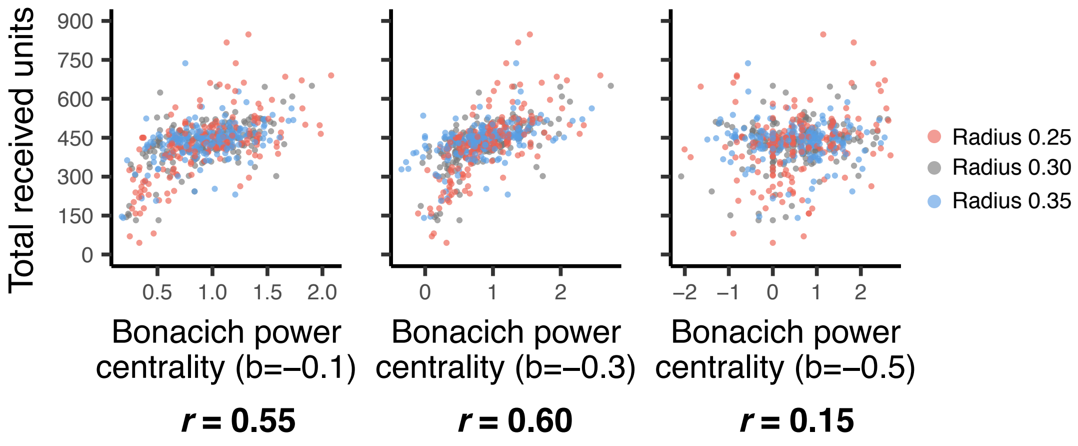

Supplementary Figure 5

Bonacich power centrality and individual wealth in sharing networks. Dots indicate the measure of Bonacich power centrality the beta value of which is -0.1, -0.3, and -0,5 separately for each subject and his or her total received units at the end of game (out of N=600 subjects who participated). Dot color indicates the network's connection radii; red for radius=0.25, gray for radius=0.30, and blue for radius=0.35. The "r" values indicate Pearson correlation coefficients (P < 0.01 for all the correlation coefficients).

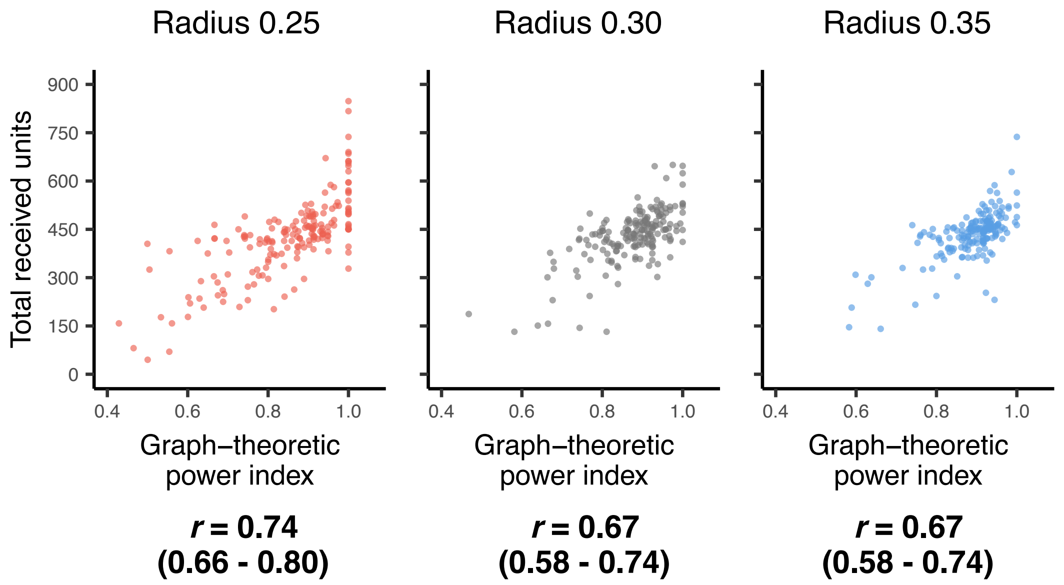

Supplementary Figure 6

Graph-theoretic power index and total receiving shares of each subject across the connection radius. The "r" values indicate the correlation coefficients with 95% confidential interval. All the correlation coefficients across the connection radius are significant (that is, the value is not zero), but the difference of correlation coefficients among connection radius is not significant. Instead, the variance of GPI in the network with radius 0.25 is larger than that in the network with radius 0.30 and 0.35 (the variance of GPI in the network with radius 0.25 = 0.0170, that with radius 0.30 = 0.0075, and that with radius 0.35 = 0.0057).

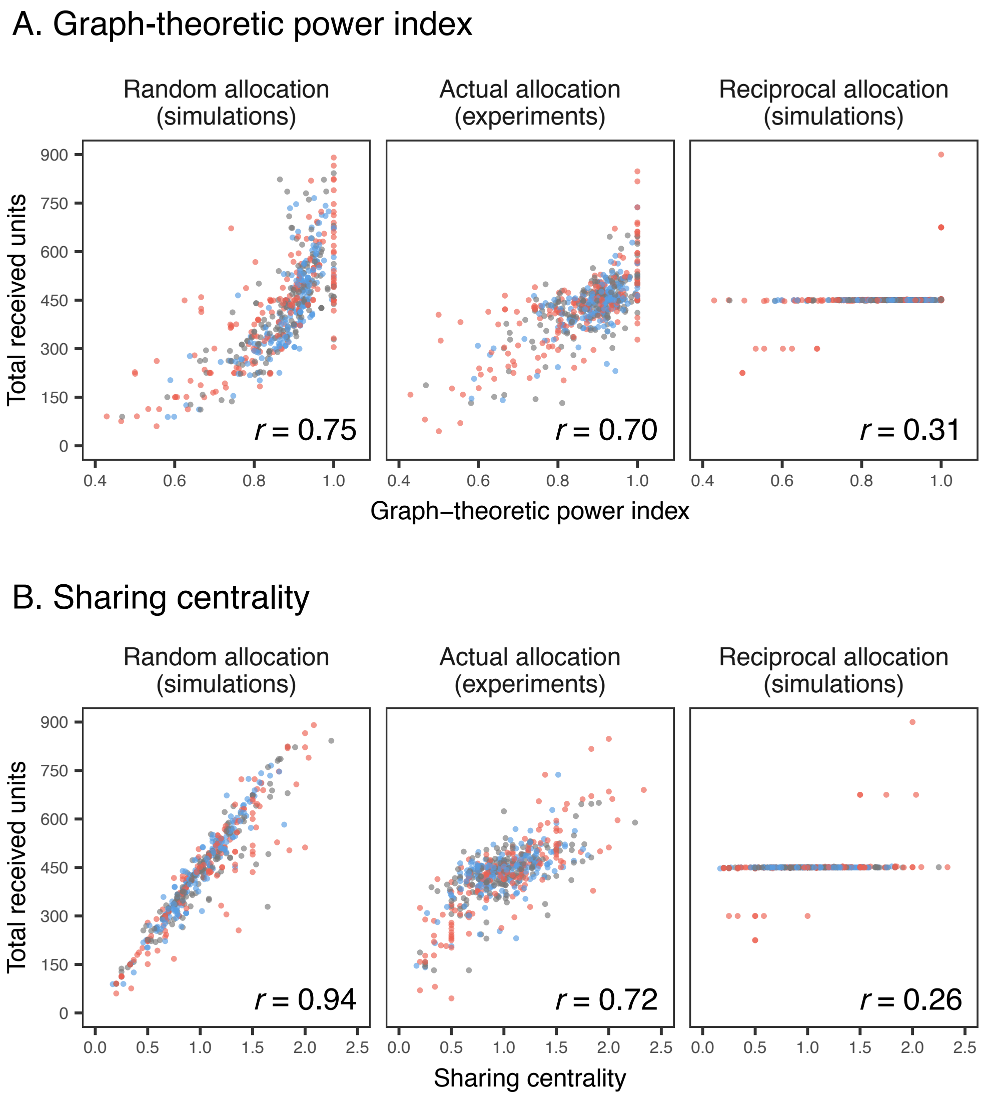

Supplementary Figure 7

The correlation between centrality measure and individual wealth, across different allocation strategies. The total received units (wealth) of "actual allocation" graphs show the experimental outcomes. Those of "random allocation" are obtained from the simulations using the null model that each individual randomly responded to neighbors while keeping the diversity of the actual allocations from the experiment. Those of "reciprocal allocation," which are also obtained from simulations, show the equilibrium of the reciprocal sharing interactions where each individual proportionally responds to their neighbors. The "r" values indicate Pearson correlation coefficients (P < 0.01 for all the correlation coefficients).

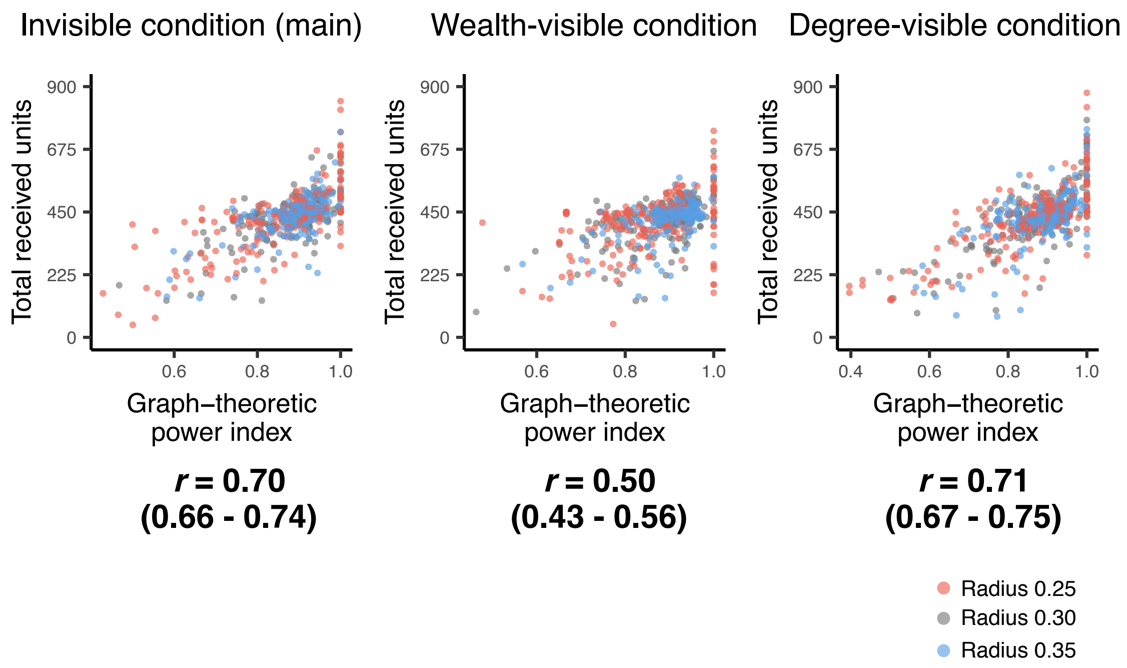

Supplementary Figure 8

Graph-theoretic power index and total receiving shares of each subject across visibility conditions. *Dots indicate the graph-theoretic power index (GPI) of each subject and his or her total received units at the end of game (out of N=600 subjects who participated). Dot color indicates the network's connection radii; red is radius=0.25, gray is radius=0.30, and blue is radius=0.35. The "r" values indicate the correlation coefficients with 95% confidential interval. All the correlation coefficients across the visibility conditions are significant. The correlation between GPI and wealth in the wealth-visible sessions is significantly smaller, compared to that in the baseline and degree-visible condition (P < 0.001).*

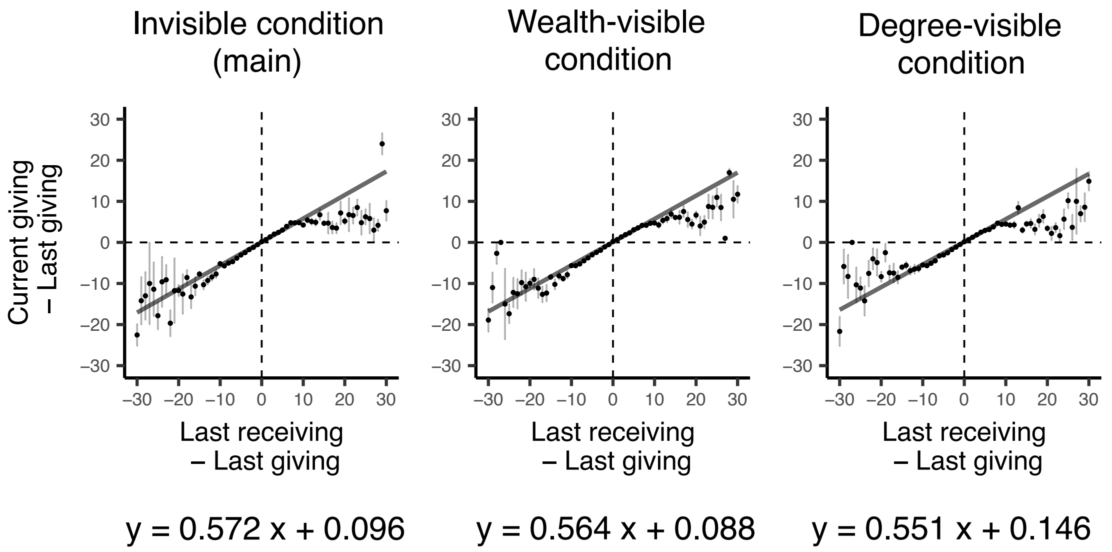

Supplementary Figure 9

Individual-level reciprocity across visibility conditions. The graphs show how much a subject increases (or decreases) their share based on the last transaction balance she or he had with each one of her or his neighbours. Dots indicate the average of actual data including all the connection radii treatments. Error bars are standard errors. Grey lines are the estimated result of regression analysis with nested random effects for round and individuals. All the estimated coefficients and intercepts are significant ($P < 0.001$). The reciprocal tendency is not different between visible treatments ($P = 0.717$ for the coefficient of wealth-visible condition and $P = 0.080$ for that of degree-visible condition, compared to that of the base condition)

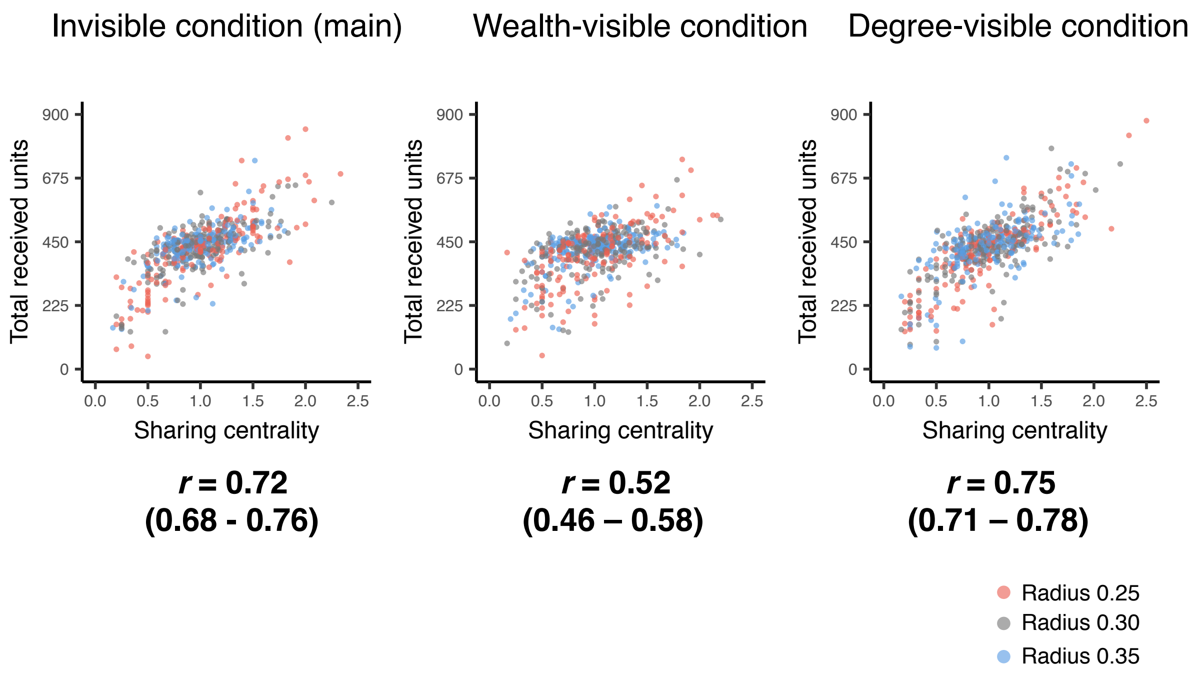

Supplementary Figure 10

Sharing centrality and total receiving shares of each subject across visibility conditions. Dots indicate the sharing centrality of each subject and his or her total received units at the end of game (out of N=600 subjects who participated). Dot color indicates the network's connection radii; red is radius=0.25, gray is radius=0.30, and blue is radius=0.35. The "r" values indicate the correlation coefficients with 95% confidential interval. All the correlation coefficients across the visibility conditions are significant. The correlation between sharing centrality and wealth in the wealth-visible sessions is significantly smaller, compared to that in the baseline and degree-visible condition (P < 0.001).

	Without visible-condition data		With visible-condition data	
	Model 1	Model 2	Model 3	Model 4
Intercept	0.296** (0.035)	0.757** (0.099)	0.265** (0.021)	0.508** (0.062)
Network density	-0.790** (0.156)	-5.291** (0.939)	-0.646** (0.089)	-3.051** (0.585)
Network density squared		10.300** (2.133)		5.559** (1.339)
Neighbors' information (Ref. = Invisible)				
Wealth visible			-0.012 (0.012)	-0.017 (0.011)
Degree visible			0.015 (0.012)	0.018 (0.011)
Number of observations	30	30	90	90
AIC	-95.1	-111.8	-288.5	-303.1

* $P < 0.05$, ** $P < 0.01$

Supplementary Table 1

The result of the statistical analysis regarding the change in Gini coefficient by network density and information visibility, estimated by linear and quadric regressions. The values in the brackets indicate the standard error of estimated coefficients.

		Estimated coefficients	Standard Error	*P* values
All	Intercept	0.057	0.013	< 0.001
	Last receiving - Last giving	0.549	0.009	< 0.001
R=0.25	Intercept	0.070	0.045	0.125
	Last receiving - Last giving	0.481	0.024	< 0.001
R=0.30	Intercept	0.143	0.039	< 0.001
	Last receiving - Last giving	0.621	0.019	< 0.001
R=0.35	Intercept	0.074	0.028	0.009
	Last receiving - Last giving	0.612	0.020	< 0.001

Supplementary Table 2

The result of the statistical analysis regarding the sharing changes of individuals to each neighbour, by the last sharing balance with the neighbour, estimated by LMM incorporating random effects for individuals and rounds (N=32764).

		Model1	Model2	Model3	Model4	Model5
Estimated coefficients	GPI	65.0*				
	Degree		51.0*			
	Closeness centrality			17.3*		
	Betweenness centrality				23.4*	
	Eigenvector centrality					15.4*
	Intercept	430.9*	440.4*	429.9*	429.9*	429.9*
AIC		6193.5	6477.8	6557.2	6541.5	6560.9
P value by the deviance difference test with Model1		-	< 0.001	< 0.001	< 0.001	< 0.001

		Model6	Model7	Model8	Model9
Estimated coefficients	Bonacich centrality (*b*=-0.1)	50.5*			
	Bonacich centrality (*b*=-0.3)		58.5*		
	Bonacich centrality (*b*=-0.5)			14.3	
	Sharing centrality				63.7*
	Intercept	430.03*	429.2*	429.7*	430.1*
AIC		6365.3	6298.5	6559.4	6143.1
P value by the deviance difference test with Model1		< 0.001	< 0.001	< 0.001	< 0.001

* $p < 0.05$

Supplementary Table 3

The result of the statistical analysis regarding the total received units of individuals, by several centrality metrics, estimated by LMM incorporating random effects for sessions (N=550). All the independent variables (i.e., centrality measures) are separately normalized with standard normal distribution for comparison.

Supplementary Methods

Recruitment procedure

A total of 1,950 unique subjects participated in our incentivized economic game experiments. Subjects were recruited using Amazon Mechanical Turk (AMT). AMT is an online labor market in which employers contract with workers to complete short tasks for relatively small amounts of money. Workers often receive a baseline payment, plus an additional bonus depending on their performance. Thus, incentivized experiments are easy to conduct using AMT. We used two baseline payments; the first corresponds to the traditional show-up fee ($2.00) and the second to the completion of the game ($2.00). This latter was necessary for deterring participants from dropping of the game due to its prolonged duration (about 40 minutes on average). Moreover, the participants received a bonus payment, which was proportional to the aggregate Internet bandwidth they received from all their neighbors throughout the game. The exchange rate of the bonus is $1.00 = 200 units.

Issues exist when running experiments online that do not arise in the traditional laboratory. For example, running experiments online naturally implies some loss of control, since the workers cannot be directly monitored as in the traditional lab; experimenters cannot be certain that each observation is the result of a single person (as opposed to multiple people making joint decisions at the same computer), or that one person does not participate multiple times (although AMT goes to great lengths to try to prevent this, and, based on IP address monitoring, it seems to happen very infrequently); and the sample of subjects in AMT experiments is restricted to people who participate in online labor markets (although most physical lab studies are restricted to college undergraduates, who are also far from representative).

Experimental setup

Our participants interacted anonymously over the Internet using customized software playable in a browser window ("Breadboard"; available at http://breadboard.yale.edu). We prohibited subjects from participating in more than one session of the experiment by using unique identifications for each subject on AMT. The experiments were conducted from March to September 2016. We conducted 10 sessions for each treatment, and each session comprised of 15 rounds where the players were making repeated decisions.

Instructions and tutorial

Below are screenshots for the initial description of the tutorial and the confirmation tests. We also show some sample screenshots of the real game. Details are provided as captions after each figure.

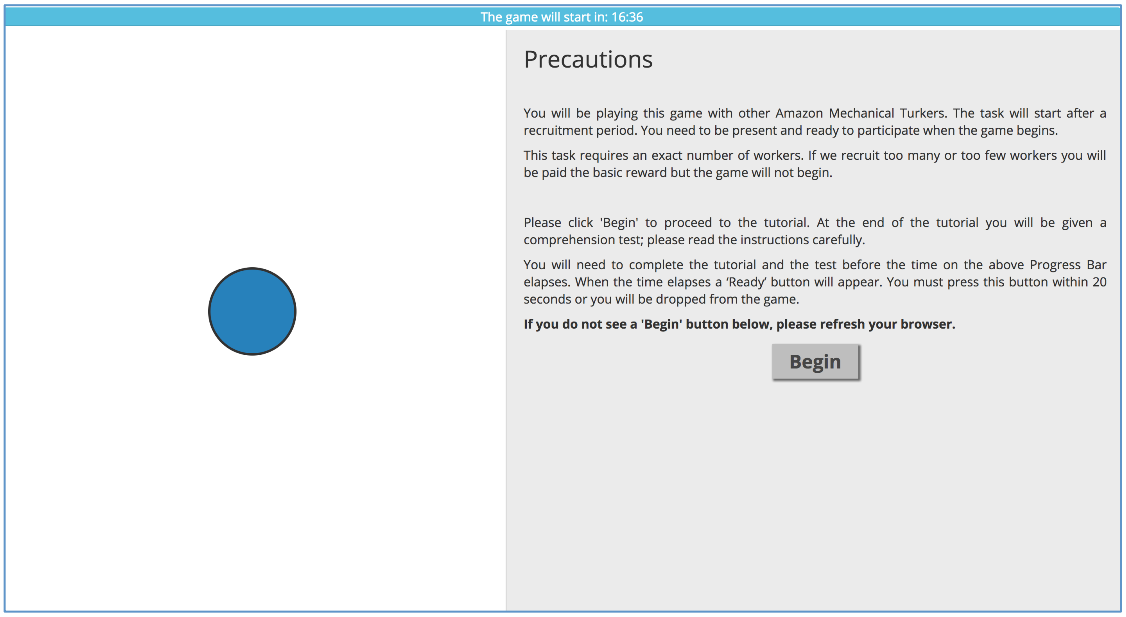

Screenshot 1. The main precautions are presented to the players.

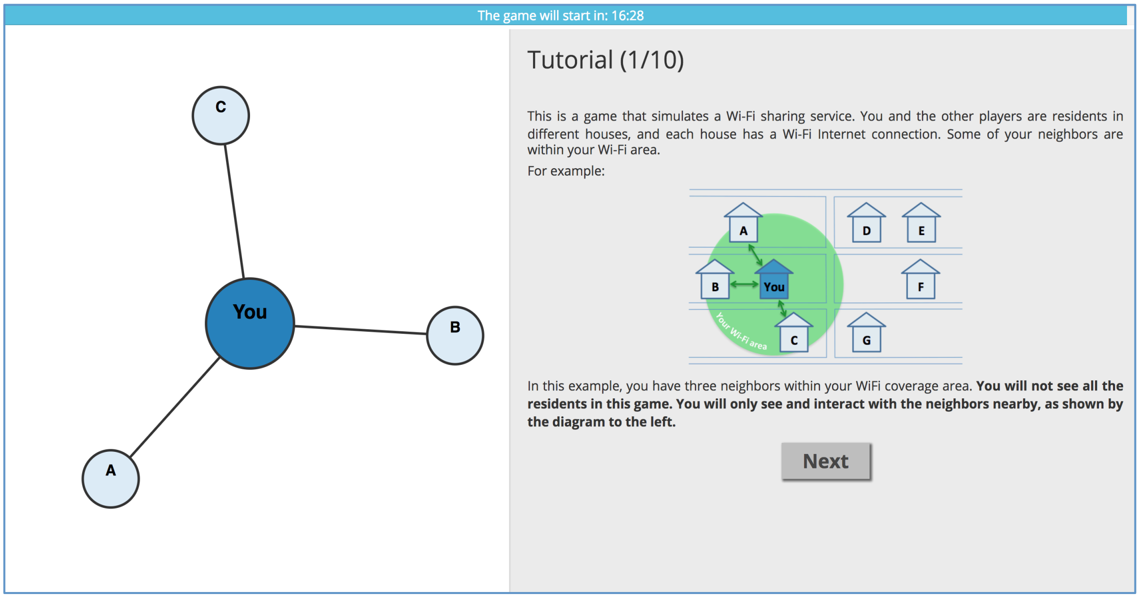

Screenshot 2. The first Tutorial screen shows a representation of the "real-world" analogy for the game, in order to increase the realism in the experiment.

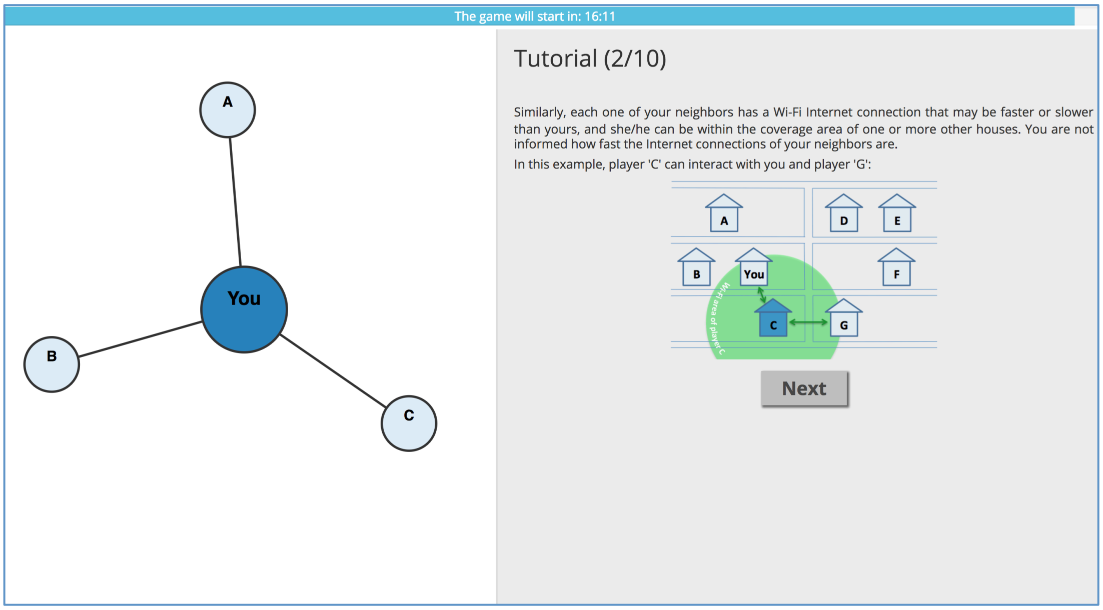

Screenshot 3. The 2nd Tutorial screen emphasizes that different houses might have different neighbors and different Internet connections. This helps to mitigate preconceptions of players about their neighbors having identical parameter values.

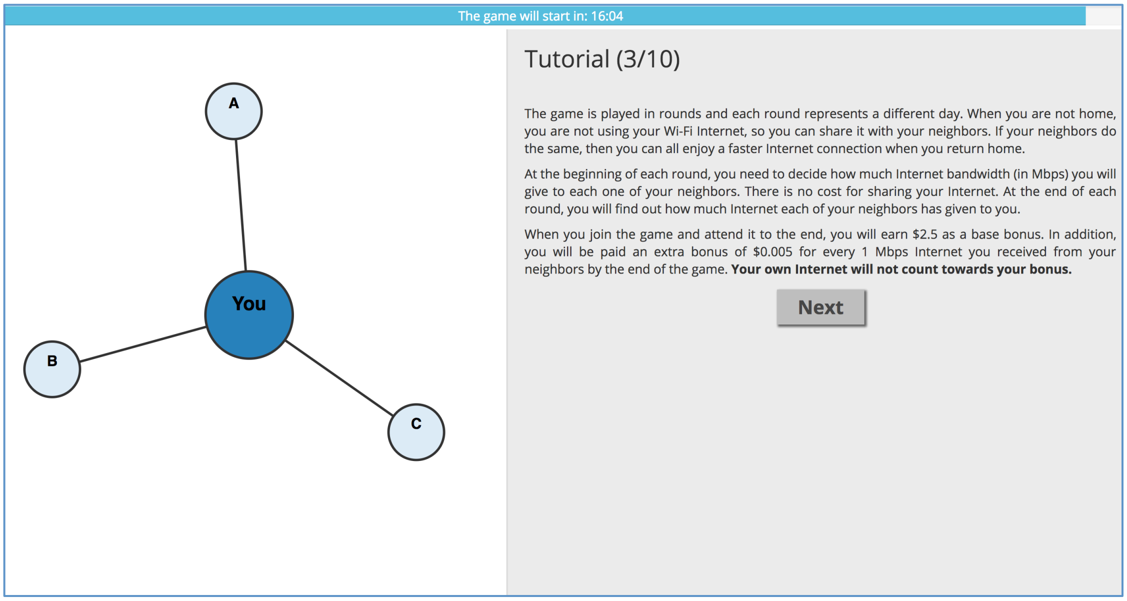

Screenshot 4. The 3rd Tutorial screen explains that the game is played in rounds; and clarifies how the bonus of each player is calculated. We put emphasis on this latter (by elaborating and using bold typeface fonts) since we need the players to comprehend the factors that affect their monetary compensation.

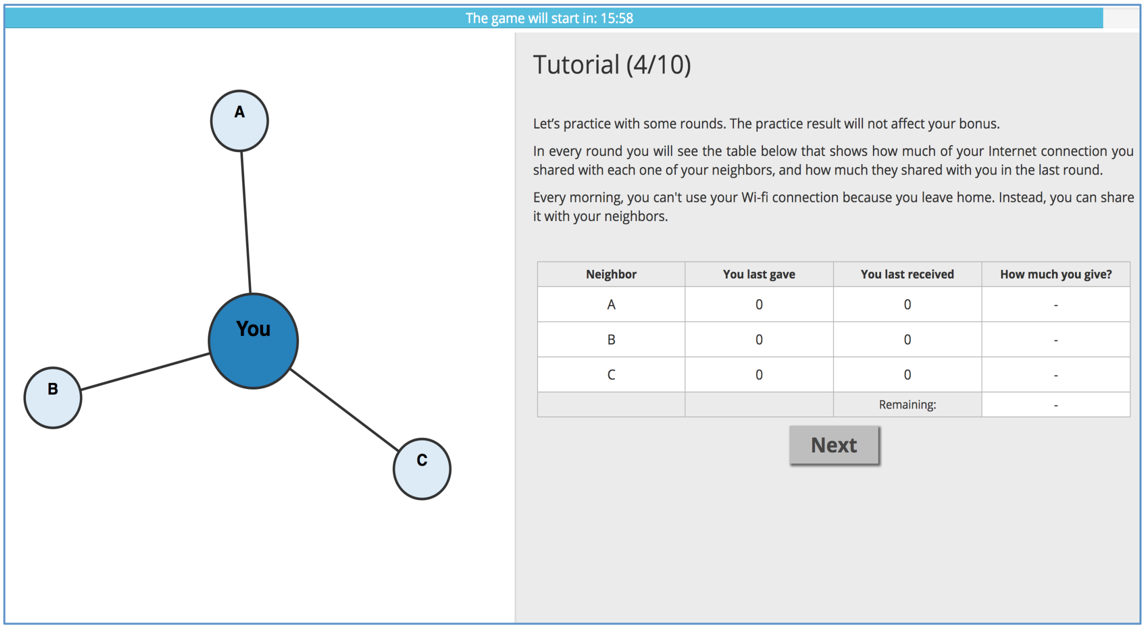

Screenshot 5. The 4th Tutorial screen initiates the practice session.

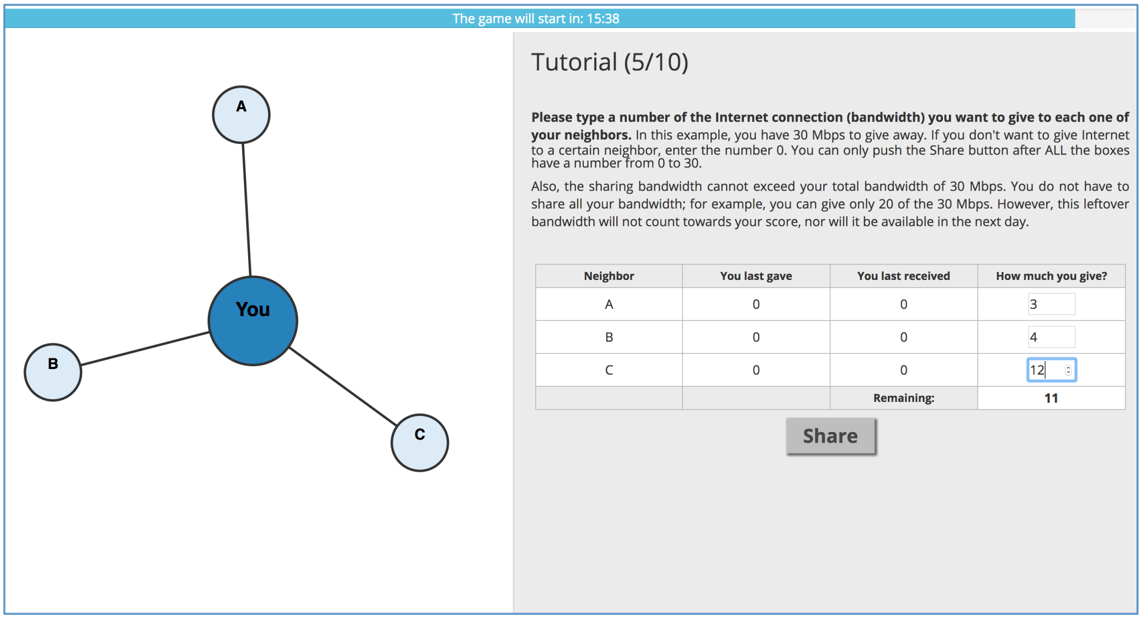

Screenshot 6. During the practice session, the players are allowed to select how much Internet they share with their neighbors. Note that we additionally provide detailed instructions and explanations about the game rules.

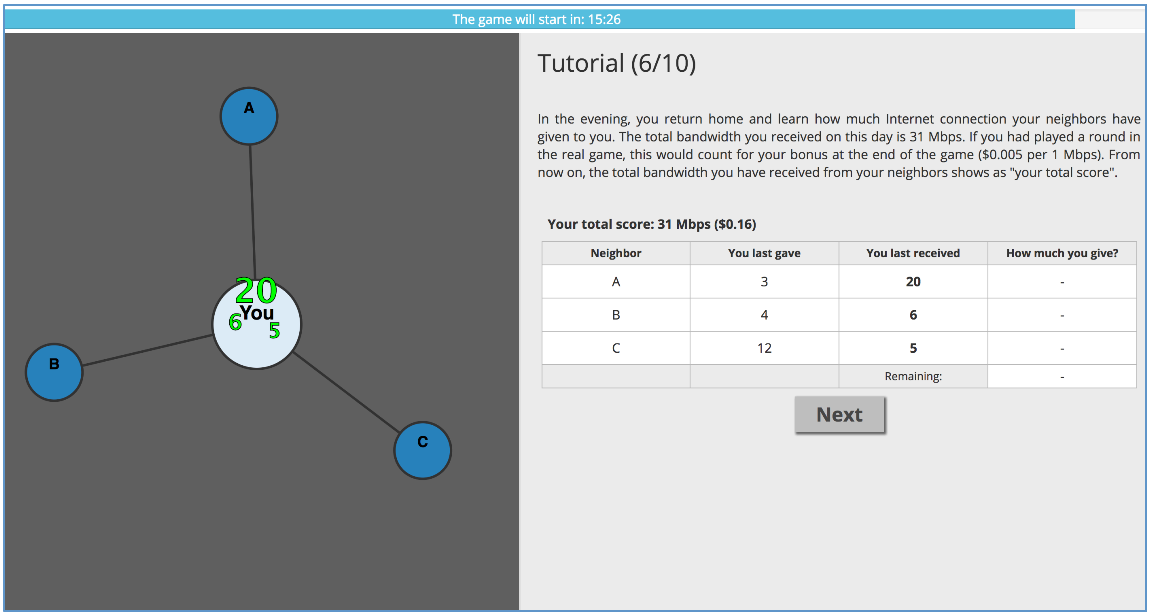

Screenshot 7. The practice session follows precisely the steps of the actual game, while it also provides additional information and explanation (right panel) to advance the players' comprehension.

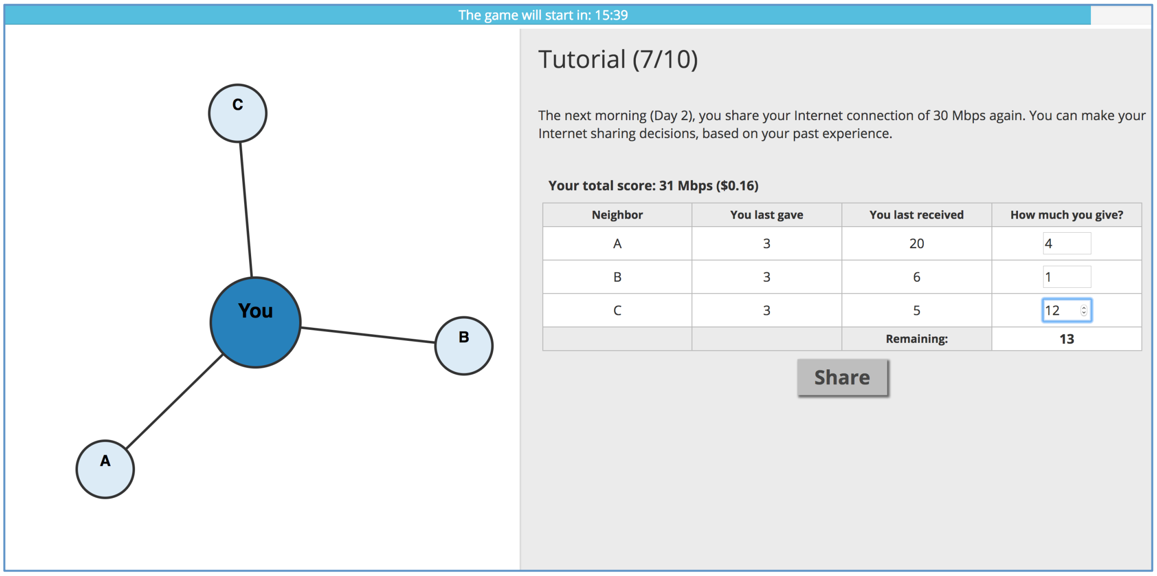

Screenshot 8. The practice session continues for another day. This is necessary for emphasizing the time dimension to the players, and the fact that their actions in one round might have impact on their game in the next round.

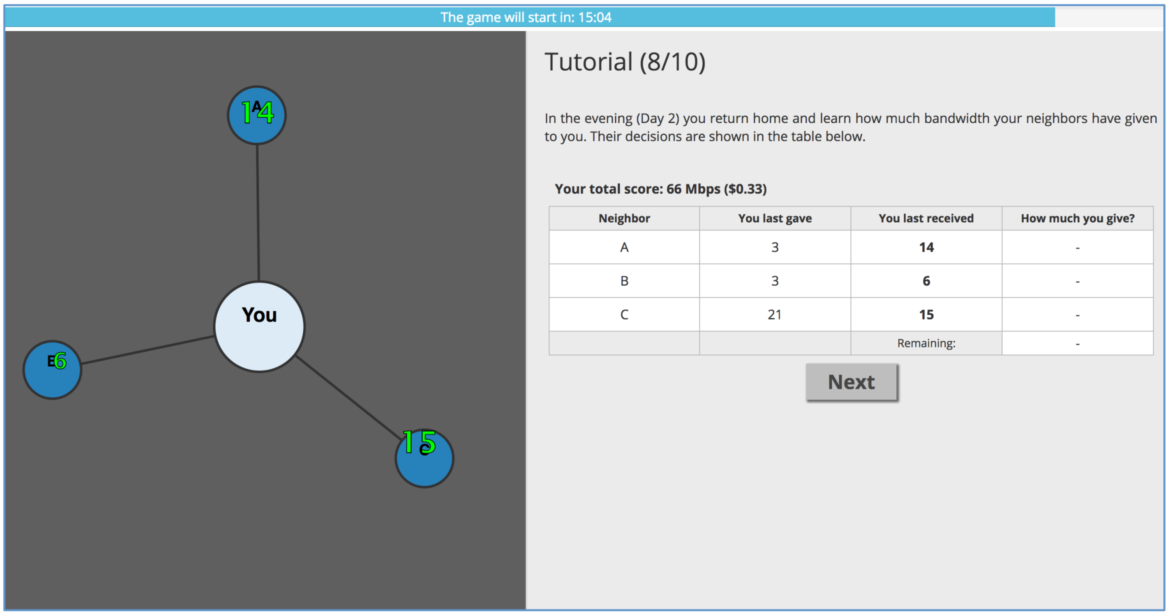

Screenshot 9. The decision of the 2nd round (day 2) are shown with an animation.

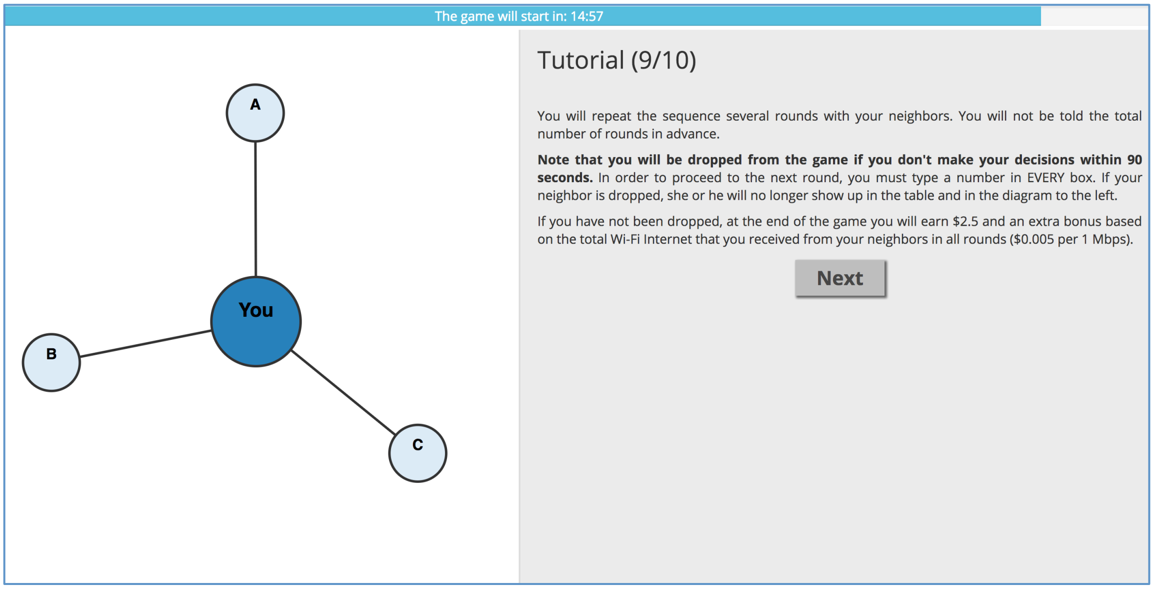

Screenshot 10. The 9th Tutorial screen concludes the practice session and gives additional guidelines, emphasizing the need for the player to make allocation decisions with the time-window of 90 seconds.

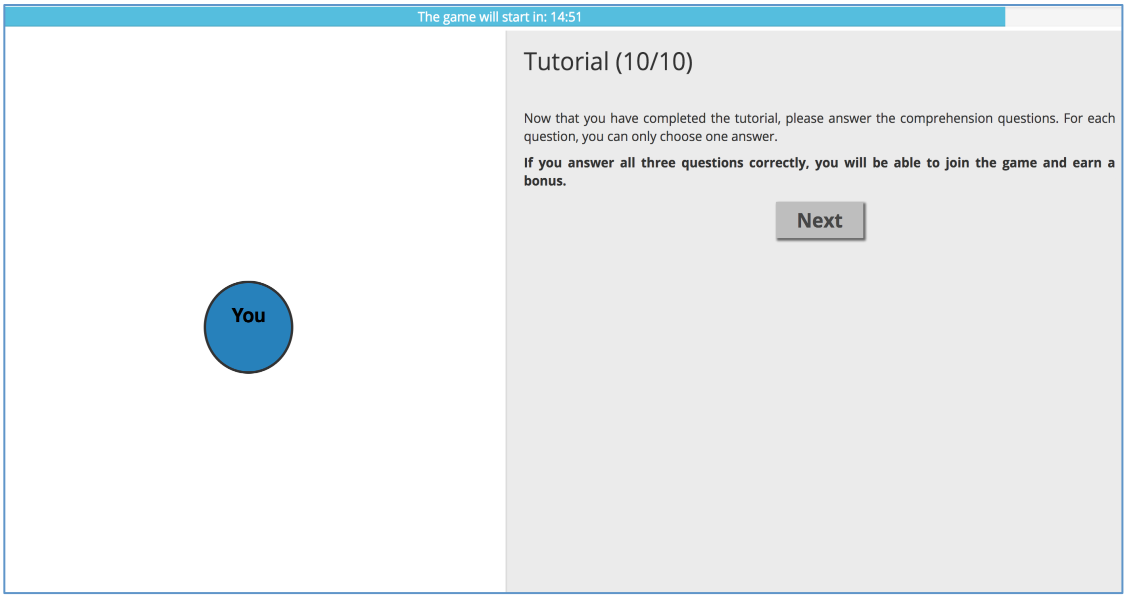

Screenshot 11. The 10th Tutorial screen explains to the players that must answer the comprehension questions, and stresses that they will be able to proceed only if answer correctly in all of them.

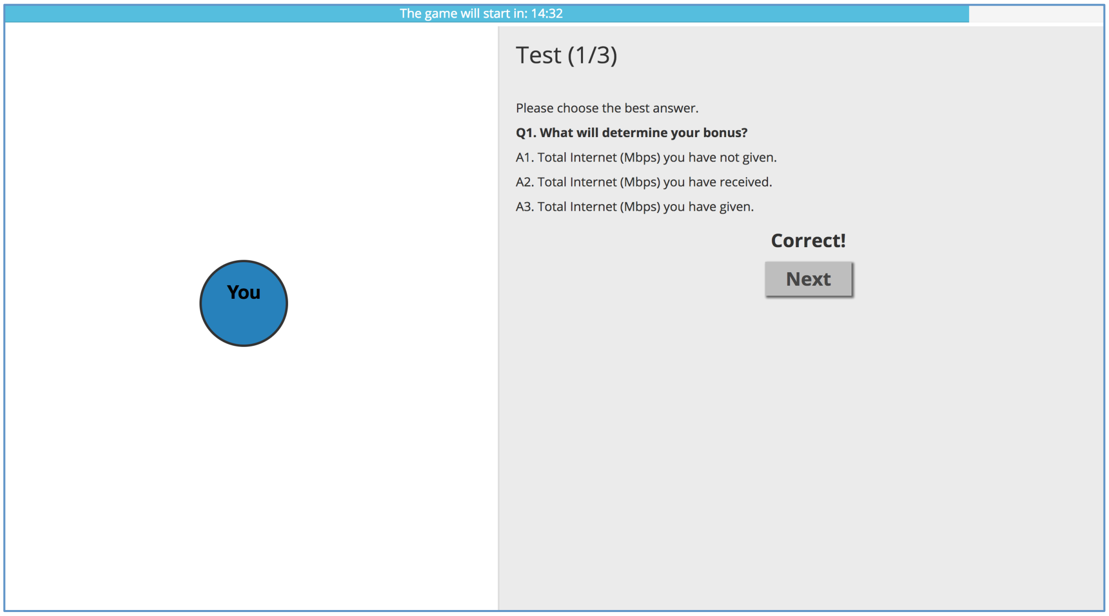

Screenshot 12. The first question asks the players what determines their bonus (correct answer is A2). This is very important as we need the players to be aware the payment rules (as this might affect their strategy). Note that for each player and each session the correct answer is allocated in different slot (i.e., not necessarily placed at A2).

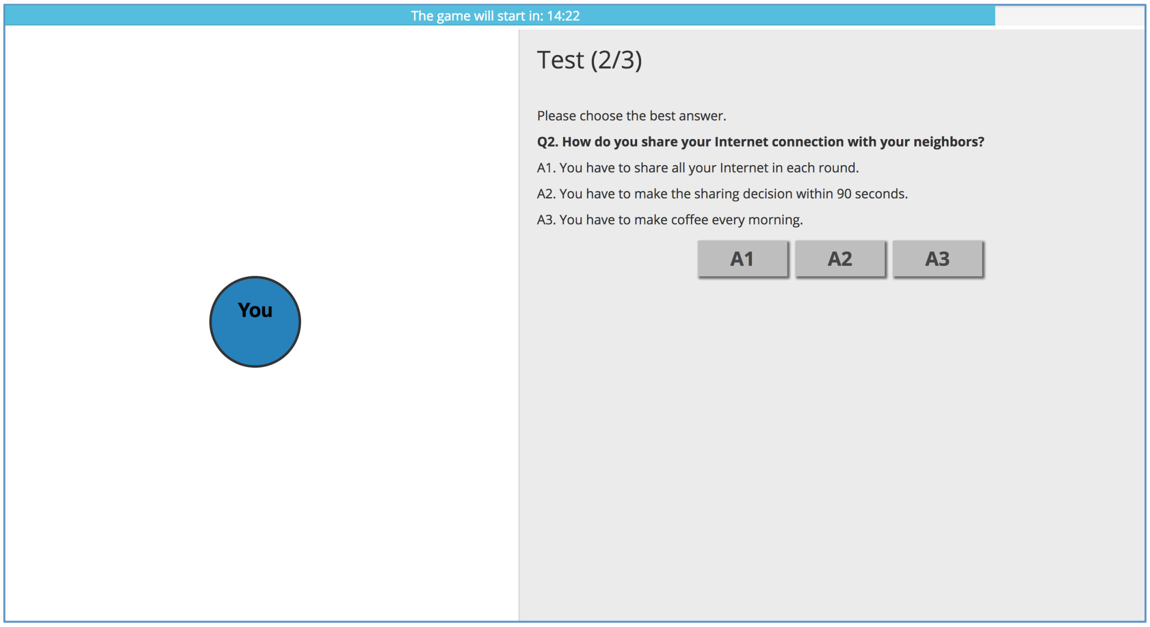

Screenshot 13. The second question asks the players about the Internet sharing rule (correct answer is A2 in this display). Clearly, players who have not understood the rules dictating how much Internet (and how) they can allocate to their neighbors should be excluded from the game.

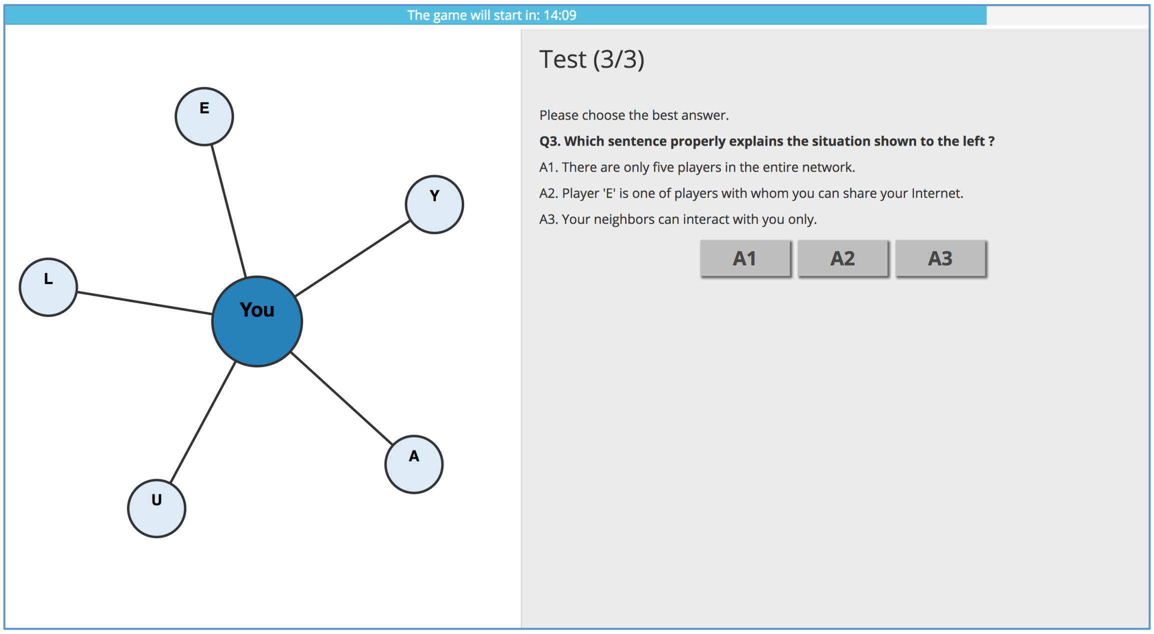

Screenshot 14. The final question is related to the graphic interface, and aims to ensure the players comprehend the visualization (and also the game rules).

We present below a sample of Tutorial screens for the information-visible treatments. The majority of the screens are the same as before, i.e., in the basic version of the game, but there are few changes.

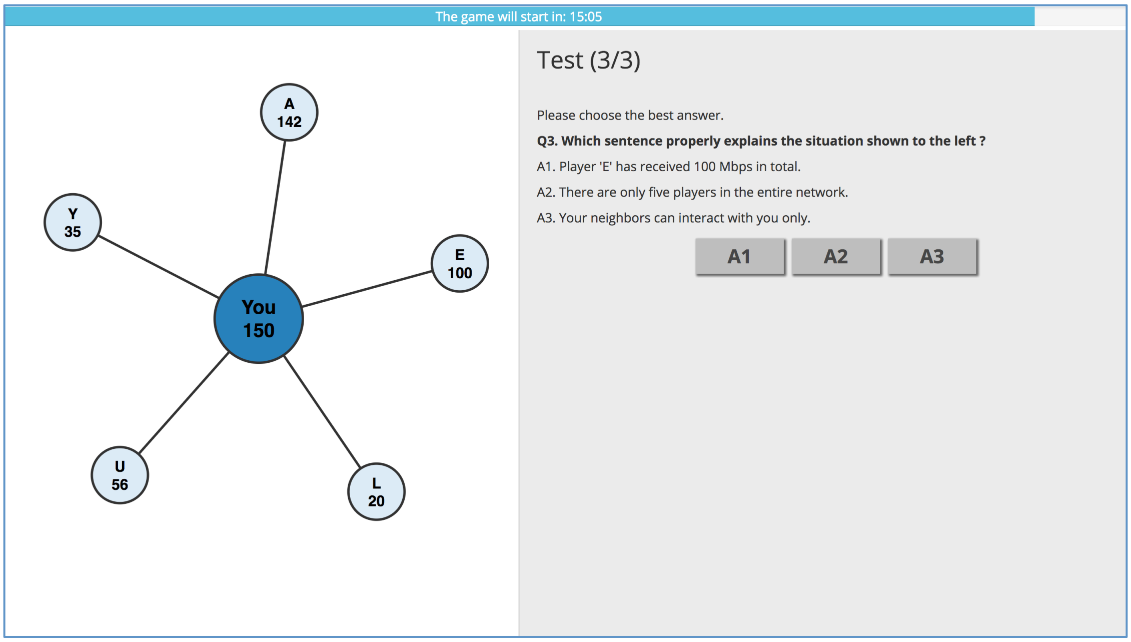

Screenshot 15. In the wealth-visibility treatment, we change accordingly the question that examines the comprehension of the graphic interface and the game rules (here, correct answer is A1)

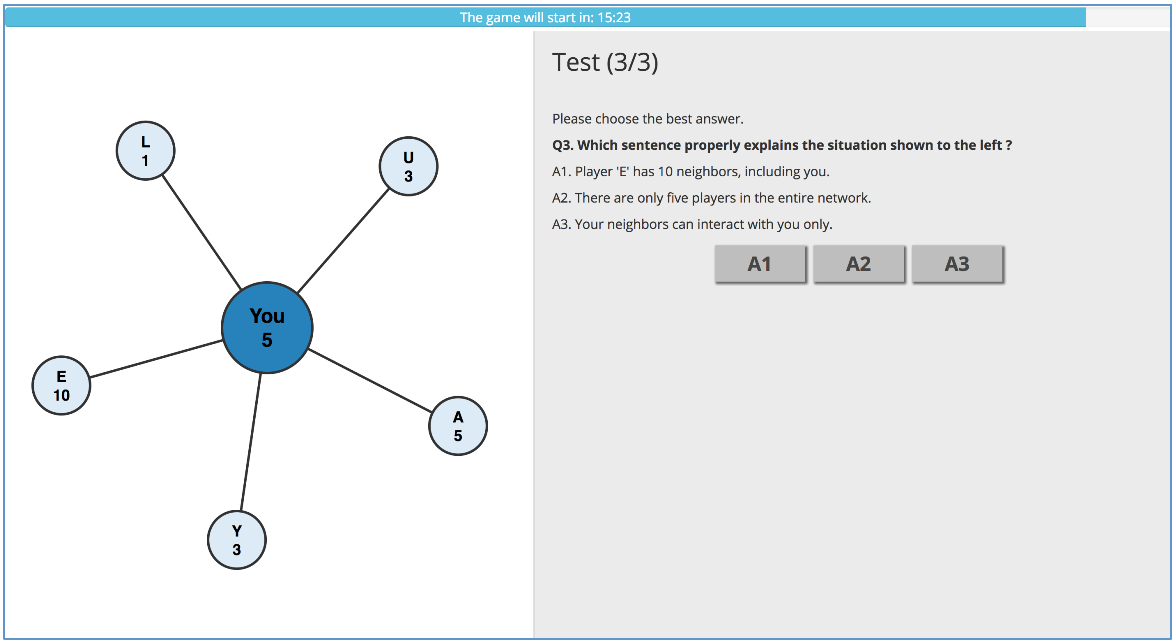

Screenshot 16. Similarly, in the degree-visibility treatment, we adapt accordingly the question (here, correct answer is A1).

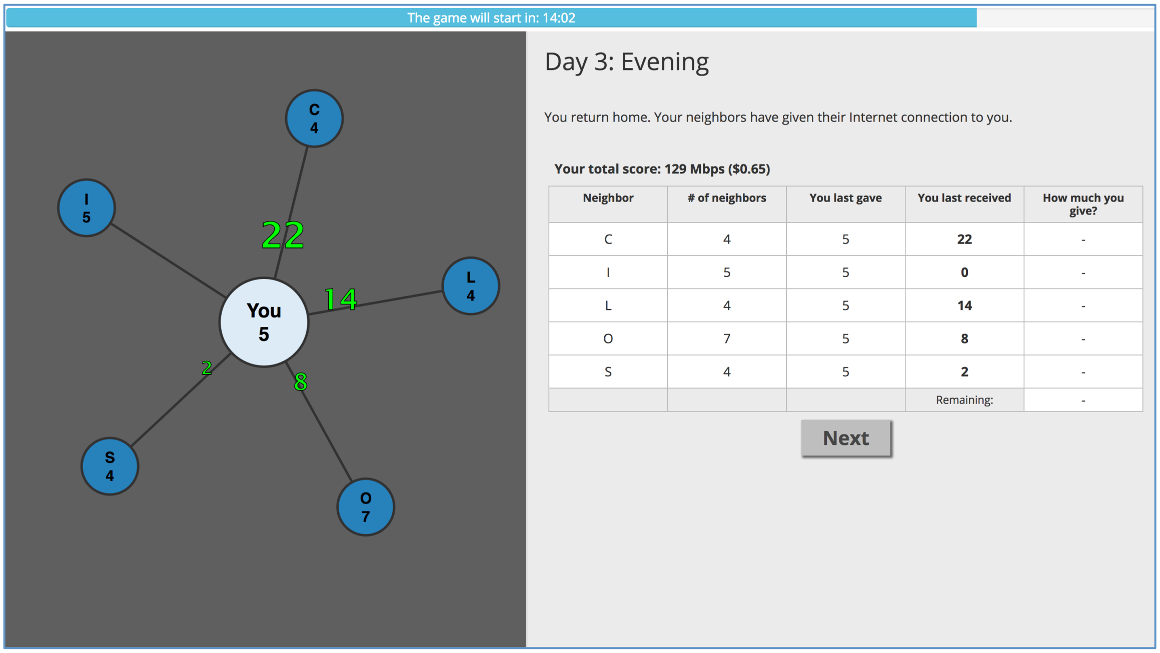

Screenshot 17. Note also that the game screens change accordingly, for both of the information-visible treatment. A column is added in the table next to the column indicating neighbours' label. Also, the same information was indicated in the nodes of the network diagram. This screenshot is for the degree-visible treatment. When the session is for the wealth-visible treatment, players would see the total score of neighbours at the time, instead of the number of neighbors.
